# Disruption of *OsPHD1*, Encoding a UDP-Glucose Epimerase, Causes JA Accumulation and Enhanced Bacterial Blight Resistance in Rice

**DOI:** 10.3390/ijms23020751

**Published:** 2022-01-11

**Authors:** Yu Gao, Xiaojiao Xiang, Yingxin Zhang, Yongrun Cao, Beifang Wang, Yue Zhang, Chen Wang, Min Jiang, Wenjing Duan, Daibo Chen, Xiaodeng Zhan, Shihua Cheng, Qunen Liu, Liyong Cao

**Affiliations:** 1 State Key Laboratory of Rice Biology and Chinese National Center for Rice Improvement, China National Rice Research Institute, Hangzhou 311401, China; 15057067488@163.com (Y.G.); xiangxiaojiao1990@163.com (X.X.); zhangyingxin@caas.cn (Y.Z.); caoyongrun93@163.com (Y.C.); nxywbf@163.com (B.W.); zhangyuerice@163.com (Y.Z.); 98481037wc@gmail.com (C.W.); jiang_min@webmail.hzau.edu.cn (M.J.); 13140230176@163.com (W.D.); cdb840925@163.com (D.C.); zhanxiaodeng@caas.cn (X.Z.); chengshihua@caas.cn (S.C.); 2 Northern Center of China National Rice Research Institute, China National Rice Research Institute, Shuangyashan 155100, China

**Keywords:** rice (*Oryza sativa* L.), lesion mimic mutant, programmed cell death, defense response

## Abstract

Lesion mimic mutants (LMMs) have been widely used in experiments in recent years for studying plant physiological mechanisms underlying programmed cell death (PCD) and defense responses. Here, we identified a lesion mimic mutant, *lm212-1*, which cloned the causal gene by a map-based cloning strategy, and verified this by complementation. The causal gene, *OsPHD1*, encodes a UDP-glucose epimerase (UGE), and the *OsPHD1* was located in the chloroplast. *OsPHD1* was constitutively expressed in all organs, with higher expression in leaves and other green tissues. *lm212-1* exhibited decreased chlorophyll content, and the chloroplast structure was destroyed. Histochemistry results indicated that H_2_O_2_ is highly accumulated and cell death is occurred around the lesions in *lm212-1*. Compared to the wild type, expression levels of defense-related genes were up-regulated, and resistance to bacterial pathogens *Xanthomonas oryzae pv. oryzae* (*Xoo*) was enhanced, indicating that the defense response was activated in *lm212-1*, ROS production was induced by flg22, and chitin treatment also showed the same result. Jasmonic acid (JA) and methyl jasmonate (MeJA) increased, and the JA signaling pathways appeared to be disordered in *lm212-1*. Additionally, the overexpression lines showed the same phenotype as the wild type. Overall, our findings demonstrate that *OsPHD1* is involved in the regulation of PCD and defense response in rice.

## 1. Introduction

Programmed cell death (PCD) generally exists in plants as an essential defense mechanism to protect against pathogen attack and for proper growth. Hypersensitive response (HR), as one of the most effective and rapid resistance reactions, is associated with the program of PCD, as well as PCD response in the absence of biotic and abiotic stress [1,2,3]. HR involves bursts of reactive oxygen species (ROS), which helps to inhibit pathogen proliferation [4]. ROS plays a vital role in pathogen defense, gene expression regulation, and cellular signaling pathways in response to multiple pathogens [5]. The accumulation of low concentrations of ROS can activate the plant defense system and participate in the cell signal transduction pathway [6]. Under adverse conditions of biotic and abiotic stress, the dynamic balance between ROS and antioxidants is shifted, and ROS accumulates in large quantities [7]. Excessive ROS accumulation causes cell and DNA damage, and will directly lead to the formation of lesions [8]. In addition to a transient burst of hydrogen peroxide production, HR is also related to callose deposition and lignification, in accumulation of phytoalexins. Furthermore, in activation of defense-related genes [9], the content of hormones also changes to a certain extent, such as jasmonic acid (JA) [10], abscisic acid (ABA) [11], and phenols [12].

PCD is a very sophisticated process involving many regulatory mechanisms. When these regulatory mechanisms are out of control, a lesion mimic phenotype will be created [13]. Most lesion mimic mutants (LMMs) are caused by PCD, and not by pathogens infection [14]. As the ideal means of dissecting HR-mediated PCD and the disease-resistant mechanism, researchers used LMMs to excavate signal pathways of PCD on a molecular basis. LMMs have been characterized in various plant species, including *Arabidopsis* [15], wheat [16], barley [17], tomato [18], groundnut [19], and rice [20]. These works show that many LMMs have activated defense responses and display a higher resistance to disease [9,20].

Over the years, a number of rice lesion mimic genes have been characterized and cloned, and LMMs have been well-documented in genetics and physiology [21]. The mechanism and pathway of rice LMMs are gradually clear, the occurrence of the lesion is caused by the generation of the in vivo cellular death, and changes of transcription factors of each physiological pathway and the product and accumulation of metabolites are generated. For example, OsCUL3a interacts with OsRBX1s to form a Cullin-Ring-like E3 ligase that targets OsNPR1 for degradation. The loss-of-function mutant *oscul3a* displays a significant increase in the accumulation of flg22-induced and chitin-induced reactive oxygen species [22]. Rice *SPL11* encodes a U-box E3 ubiquitin ligase which also negatively regulates cell death and immunity in a 26S proteasome-dependent manner [23]. SPL33, an eEF1A-like protein, contains a nonfunctional zinc-finger domain and three functional EF-Tu domains, involved in the regulation of PCD [24]. Furthermore, *SPL35*, which encoded a CUE-domain protein, may be involved in ubiquitination and vesicular trafficking pathways [9]. *OsNBL3* encodes a mitochondrion-localized pentatricopeptide repeat (PPR) protein. Furthermore, it is involved in splicing nad5 intron 4, which is essential for mitochondrial development and functions, and its disruption causes the lesion mimic phenotype [25]. OsLOL1, a C2C2-type zinc finger protein, promotes gibberellin (GA) biosynthesis and affects seed germination [26]. *OsSSI2* is involved in the negative regulation of defense responses in rice, and the induction of SA-responsive genes is likely responsible for enhanced disease resistance in OsSSI2-kd rice plants [27]. *OsHPL3* regulates the specific defense response of rice to different invaders by affecting the content of JA and other volatiles, and disease resistance is, therefore, changed [10,28]. With the discovery of LMMs and the characterization of genes, the molecular mechanisms of plant disease resistance are gradually being improved, but there are many underlying deeper mechanisms that have not been fully understood.

UDP-galactose (UDP-Gal) is essential for the biosynthesis of heteroxylans, glycoproteins, and glycolipids. UDP-galactose glucose epimerases (UGEs) have been reported to be involved in the bioconversion of UDP-Gal and UDP-glucose (Glc) [29]. Five UGE isoforms have been identified in *Arabidopsis*, including AtUGE1, AtUGE2, AtUGE3, AtUGE4, and AtUGE5. They participate in cell wall biosynthesis and pollen development, and some of them respond to environmental stress [30]. Four genes encoding UGEs have been identified in rice, OsUGE1 participates in cell wall carbohydrate partitioning under low nitrogen [31], while OsUGE2 participates in cellulose biosynthesis and cell wall assembly [32], and *OsPHD1* plays an important role in photosynthetic activity. However, whether UGEs participate in the regulation of cell death and disease resistance is largely unknown.

In the present study, we identified and characterized a rice LMM, named *lm212-1*, which exhibited lesions on plants from the seedling stage. The gene we located, *OsPHD1*, encodes a UGE, influences the amounts of UDP-Glc and UDP-Gal, and eventually affects the lipid composition [33]. In terms of the mutant, ROS balance was destroyed, cell death occurred, the structure of the chloroplast was damaged, and the resistance to bacterial blight diseases was enhanced, accompanied by an increase in contents of jasmonic acid (JA) and methyl jasmonate (MeJA). Our findings revealed that disruption of *OsPHD1* can result in the formation of lesions, and is involved in the regulation of PCD and defense response in rice.

## 2. Results

### 2.1. Phenotypic Characterization of the lm212-1 Mutant

**Figure 1:**
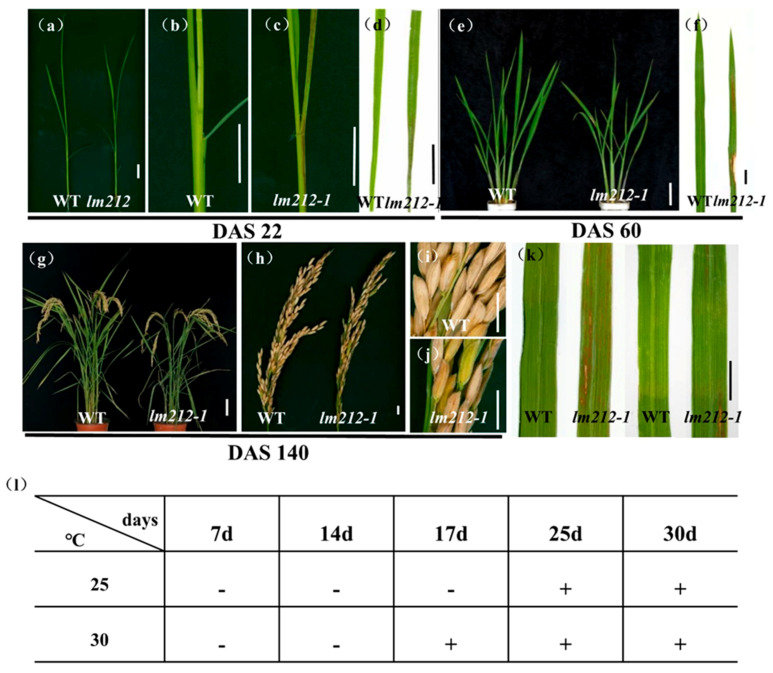
Phenotypes of WT and the *lm212-1* mutant. (**a**) Plants at the seedling stage (DAS22). (**b**,**c**) Stem and leaf sheath at the seedling stage (DAS22). (**d**) Leaves at the seedling stage (DAS22). (**e**) Plants at the tillering stage (DAS60). (**f**) Leaves at the tillering stage (DAS60). (**g**) Plants at the mature stage (DAS140). (**h**–**j**) Phenotypes of panicle at the mature stage (DAS140). (**k**) Leaf blades of WT and *lm212-1* at the tillering stage. (**l**) Temperature treatment assay of the *lm212-1* mutant at 25 °C or 30 °C. +, lesions appear; −, normal leaves. Scale bars (**a**–**d**,**f**,**j**,**k**), 1 cm; Scale bars (**e**,**g**), 10 cm.

The *lm212-1* mutant was obtained from an ethyl methane sulfonate (EMS)-induced mutant of a *japonica* rice variety JiaHe 212. Lesions were first observed in the seedling stage (22 days after sowing (DAS22) (Figure 1a), with the first appearing on the sheath and leaves as brown spots (Figure 1b–e). At the tillering stage (DAS60), lesions on the leaves were aggravated (Figure 1e,f), becoming longer and wider. The lesions remained at the maturity stage (DAS140) (Figure 1g,h), sometimes appearing in seeds (Figure 1i,j). The facts showed that the lesion development was dependent on the growth process of rice plants. At the same time, the tiller number, plant height, flag leaf length, seed setting rate, filled grain per panicle, and 1000-grain weight of the *lm212-1* mutant were lower than the wild type (WT). In the meantime, grain width and grain length were obviously smaller in the *lm212-1* mutant (Figure S1).

In some LMMs, light and temperature can induce the formation of lesions mimic symptoms [34]. To test whether the lesions of the *lm212-1* mutant were induced by light and temperature, we conducted shading and temperature treatments at the tillering stage. In the shading experiment, we covered the 3-m aluminum foil at the middle of the second leaves. After a week, in the *lm212-1* mutant, the shaded areas were covered with aluminum foil, and lesions were not observed (Figure 1k). In the temperature experiment, the *lm212-1* seeds were cultivated in a temperature treatment incubator at 30 °C or 25 °C. The lesion phenotype appeared at 30 °C after 17 days, while the 25 °C treatment did not cause any of it until about 25 days (Figure 1l). Those results all indicate that the formation of the lesions was regulated by both light and temperature.

### 2.2. Photosynthetic Changes and Destruction of Chloroplast in the lm212-1 Mutant

**Figure 2:**
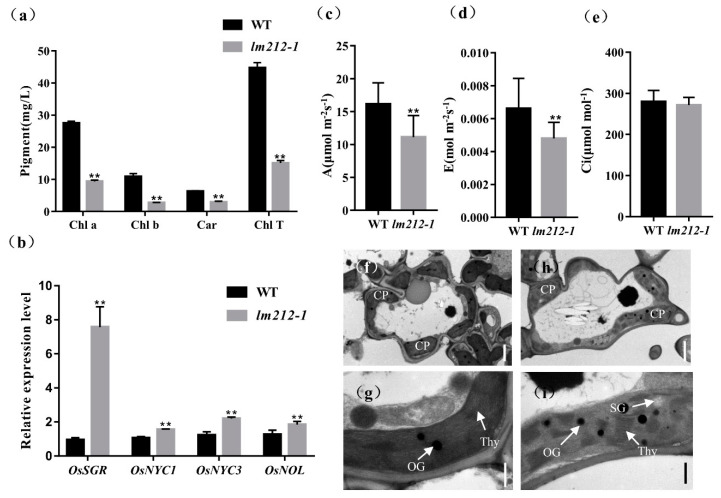
Determination of chlorophyll (Chl) content, photosynthetic rate, expression of genes associated with chloroplast degradation, and ultrastructural analysis of chloroplasts. (**a**) The Chl content at the tillering stage. (**b**) Expression analysis of chloroplast degradation-related genes in the WT and *lm212-1* leaves. (**c**) Net photosynthetic rate (A) at the heading stage. (**d**) Transpiration rate (E) at the heading stage. (**e**) Intercellular CO_2_ concentration (Ci) at the heading stage. (**f**–**i**) Transmission electron microscopy of chloroplasts in WT leaves and *lm212-1* leaves. (**f**,**g**), WT leaves. (**h**,**i**) *lm212-1* leaves. Cp, chloroplast; SG, starch granule; Thy, thylakoid; OG, osmiophilic granule. Scale bar: 2 µm in (**f**,**h**), 0.5 µm in (**g**,**i**). ** *p* < 0.01 by the Student’s *t*-test. Data represent means ± SD of three biological replicates.

We noticed that the leaf color of the *lm212-1* where lesions exist was different from that of the WT. Thus, to characterize the color abnormalities of the leaves in the *lm212-1* mutant, we measured the chlorophyll (Chl) content at the tillering stage. The results showed that the carotenoid (Car), Chl a, and Chl b contents of the *lm212-1* leaves were decreased compared to the WT leaves. Simultaneously, the total Chl content of the *lm212-1* leaves also significantly decreased compared to the WT (Figure 2a). On the other hand, the quantitative real-time PCR (qRT-PCR) of the senescence-related genes revealed that the expression level of *OsNYC1*, *OsNYC3*, *OsNOL*, and *OsSGR* in the *lm212-1* were significantly up-regulated (Figure 2b). The results indicated that the appearance of lesion spots caused a decrease in photosynthetic pigments and the chloroplast degradation.

So as to explore whether the photosynthetic capacity has changed in the *lm212-1* mutant or not, the photosynthetic parameters of the *lm212-1* and WT flag leaves were measured at the heading stage. The results showed that the net photosynthetic rate € and transpiration rate (A) of the *lm212-1* mutant were significantly lower than the WT, and there was no significant difference in the intercellular CO_2_ concentration (C) (Figure 2c).

In order to determine whether the chloroplast structure in the *lm212-1* mutant was affected, we used transmission electron microscopy (TEM) to study the ultrastructure of the chloroplast. In the WT leaves, the chloroplast structure was normal, and the thylakoids were arranged in order, with rich lamellae and a small number of osmiophilic bodies (Figure 2f,g). Where the leaves of the *lm212-1* mutant had lesions, the chloroplast structure was abnormal, the chloroplast envelope was breaking and becoming lighter in color. Moreover, the lamellar structure of chloroplasts began to collapse, and the number and size of osmiophilic bodies in the mutant chloroplasts significantly increased. The number of starch granules in *lm212-1* leaves was higher than the WT (Figure 2h,i).

The above results indicate that lesions appearance affected photosynthesis. The lower level of chlorophyll content probably reduce photosynthetic capacity, resulting in poor agronomic traits.

### 2.3. Cell Death and H_2_O_2_ Accumulation in the lm212-1 Mutant

**Figure 3:**
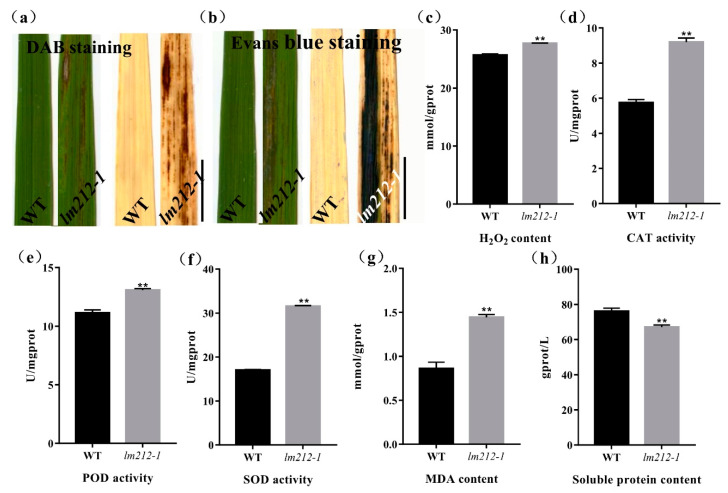
Histochemical staining and physiological detection. (**a**) DAB staining in leaves of WT and *lm212-1* at the tillering stage. (**b**) Evans blue staining in leaves of WT and *lm212-1* at the tillering stage. Scale bars, 1 cm. (**c**–**h**) Measurements of activities and contents in WT and lm212-1. (**d**) CAT activity (**e**) POD activity, (**f**) SOD activity, (**e**) H_2_O_2_ content, (**g**) MDA content, (**h**) soluble protein content. ** *p* < 0.01 by the Student’s *t*-test. Data represent means ± SD of three biological replicates.

To examine the biochemical mechanisms of the lesion mimic in the *lm212-1* mutant, biochemical stains were used to investigate the WT and the *lm212-1* mutant, including Evans blue staining for cell death and 3,3-diaminobenzidine (DAB) staining for H_2_O_2_ accumulation. DAB staining showed that the dyed brown leaves with lesions were deeper in the *lm212-1* mutant when the brown dyeing could not be observed on leaves of the WT (Figure 3a). Evans blue staining showed that, in the *lm212-1* mutant, there was clear blue coloration in the leaves, and the staining was deeper on the leaves with lesion spots compared to the WT leaves (Figure 3b). To further explore the H_2_O_2_ accumulation in the leaves of the *lm212-1* mutant, we measured related physiological indicators, including the concentration of H_2_O_2_ and the enzyme activity of catalase (CAT), superoxide dismutase (SOD), and peroxidase (POD). The concentration of H_2_O_2_ was obviously higher in the *lm212-1* than in the WT. In addition, all the CAT, SOD, and POD ezyme activity of the *lm212-1* was significantly higher than that of the WT (Figure 3c–f). To determine the extent of membrane damage, we detected the contents of lipid oxidation product malondialdehyde (MDA) and soluble protein. Compared with the WT, MDA content was also significantly increased in the *lm212-1*, while the soluble protein content was significantly decreased (Figure 3g,h), which clearly reflected the degree of cellular damage. Overall, our results suggested that ROS-scavenging system is disordered and that cell damage caused by oxidation may result in the lesion mimic phenotype of *lm212-1.*

### 2.4. Altered ROS Generation Induced by Pathogen-Associated Molecular Pattern (PAMP) Treatments

**Figure 4:**
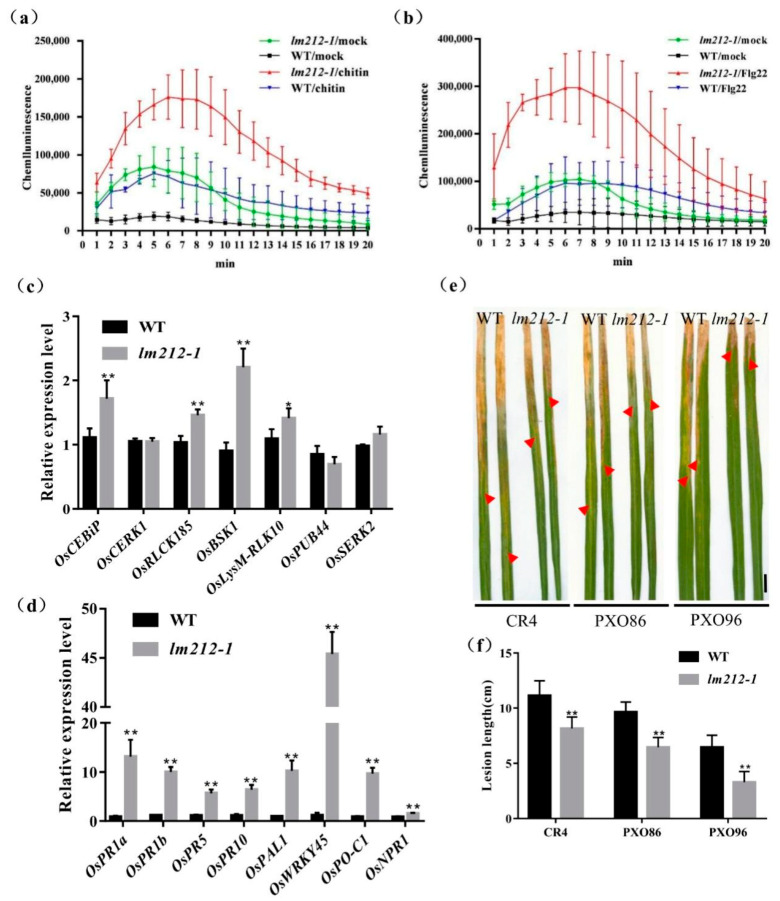
Identification of disease resistance. (**a**–**c**) ROS accumulation after mock (ddH_2_O), flg22, and chitin treatments and expression levels of genes related to PTI response pathway. (**a**) ROS accumulation dynamics of WT, *lm212-1* after chitin treatment. (**b**) ROS accumulation dynamics of WT, *lm212-1* after flg22 treatment. (**c**) Expression analysis of PTI response-related genes in WT and *lm212-1* at the tillering stage. (**d**) Expression analysis of defense response-related genes in WT and *lm212-1.* * *p* < 0.05 and ** *p* < 0.01 by the Student’s *t*-test. Data represent means ± SD of three biological replicates. (**e**,**f**) Detection of the resistance to bacterial blight *Xoo*. (**e**) Leaves of WT and *lm212-1* after inoculation with CR4, PXO86, and PXO96. The red arrows represent the lesion length. Scale bars, 1 cm. (**f**) Lesion length of WT, *lm212-1.* ** *p* < 0.01 by the Student’s *t*-test. Data represent means ± SD of ten biological replicates.

To further explore the molecular mechanism of the immune response in the *lm212-1* mutant, we used the chemiluminescence method to measure the ROS dynamics after bacterial flagellin epitope (flg22) and hexa-N-acetyl-chitohexaose (chitin) treatment. In both the mutant and the WT leaves, ROS bursts were observed after treatment. Maximum accumulation of ROS in the *lm212-1* mutant after chitin treatment is about 2.3 times that of the WT (Figure 4a), and 3.4 times after flg22 treatment (Figure 4b). Overall ROS accumulation levels were relatively higher, and the rate of ROS production was faster in *lm212-1*, even in the mock (with the ddH_2_O measurement). Furthermore, its basal ROS levels were relatively higher in the *lm212-1*. Corresponding to the difference in ROS accumulation after treatment between WT and mutant, the expression level of genes (*OsCEBiP*, *OsCERK1*, *OsRLCK185*, *OsBSK1*, *OsLysM-RLK10*, *OsPUB44*, and *OsSERK2*) involved in PAMP-triggered immunity (PTI) response was significantly up-regulated, except for *OsPUB44* (Figure 4c). These results indicated that the ROS signaling pathway of PTI is enhanced in the *lm212-1* mutant.

### 2.5. The Defense Response in the Mutant lm212-1 Is Activated and the Resistance Is Enhanced

Typical lesion mimic mutants will show increased resistance to pathogenic bacteria, as many LMMs are often accompanied by the activation of defense responses. In order to detect the expression changes of defense response-related genes in the *lm212-1* mutants, the expression of several defense response-related genes were measured including *OsPR1a*, *OsPR1b*, *OsPR5*, *OsPR10*, *OsPAL1*, *OsWRKY45*, *OsPO-C1*, and *OsNPR1*. The expression of defense-related genes in the *lm212-1* mutant was significantly up-regulated compared with WT (Figure 4d). At the same time, to examine the resistance of *lm212-1* to bacterial blight, three bacterial blight *Xoo* strains (CR1, PXO86, and POX96) were used to inoculate the leaves at the tillering stage by the leaf cutting method. After 14 days, the result showed that the WT was more susceptible to CR1, PXO86, and PXO96 (Figure 4e). Compared with the WT, the lesion length of the mutant was significantly shorter, which indicated that the mutant had enhanced resistance to bacterial blight (Figure 4f). Thus, we speculate that the defense responses activated in *lm212-1* enhanced disease resistance.

### 2.6. Map-Based Cloning of the OsPHD1

**Figure 5:**
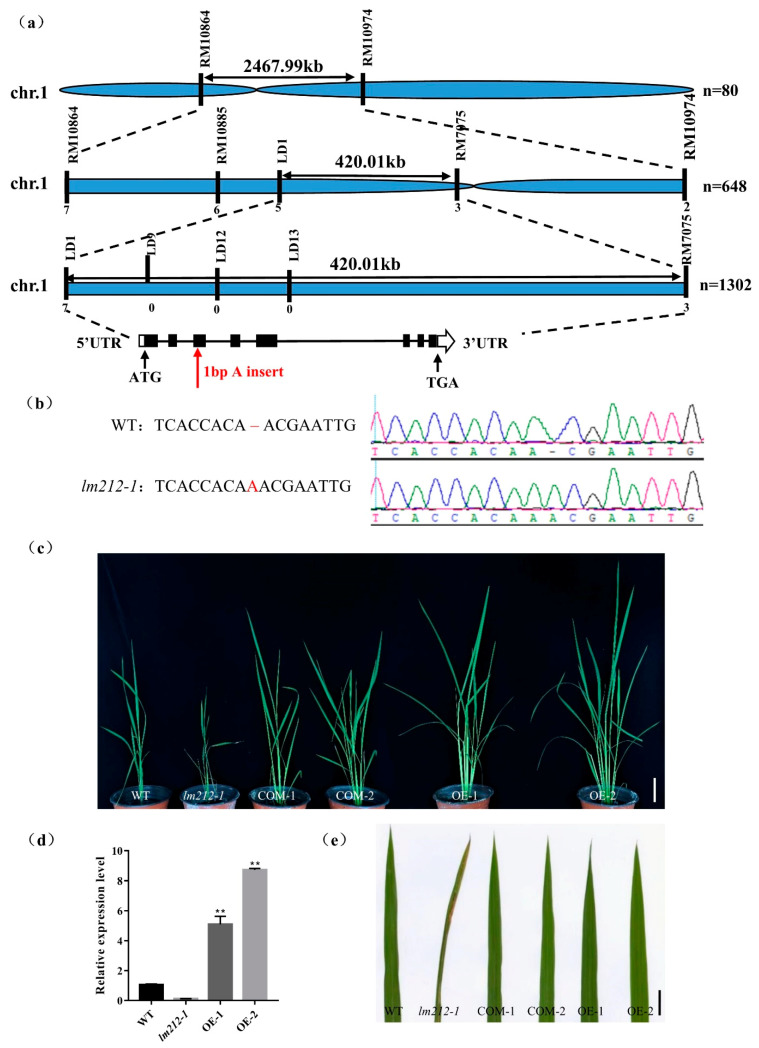
Map-based cloning and identification of *OsPHD1*. (**a**) *OsPHD1* was fine-mapped to a 420-kb genomic region between markers LD1 and RM7075. (**b**) Sequencing comparison of mutation sites in WT and the *lm212-1* mutant; an A insertion was identified, resulting in an early termination of translation. (**c**,**e**) The phenotype was completely recovered in COM-1/2 and OE-1/2. Scale bar 10 cm in (**c**), 1 cm in €. (**d**) Expression levels of *OsPHD1* in WT, *lm212-1*, and OE plants at the tillering stage. ** *p* < 0.01 by the Student’s *t*-test. Data represent means ± SD of three biological replicates.

To explore the molecular mechanisms controlling the *lm212-1* lesion phenotype, we performed a cross between the *lm212-1* and Zhonghui8015, whereby all F_1_ individuals exhibited a similar phenotype to the WT. In the F_2_ progenies, the WT and mutant phenotypes fitted well with the Mendelian segregation ratio (3:1) (Table S2), which implies that the *lm212-1* phenotype was controlled by a single recessive gene. To map the mutated gene, we selected polymorphic simple sequence repeat (SSR) markers distributed across the 12 chromosomes for linkage analysis with 80 recessive individuals from F_2_ population. The result showed that *OsPHD1* was mapped on chromosome 1 between the SSR markers RM10864 and RM10974. We used three SSR markers and newly developed polymorphic InDel markers for genotyping 648 recessive individuals and obtained the preliminary linkage interval within 420-kb between LD1 and RM7075. For the fine mapping of *OsPHD1*, five SSR/InDel markers were developed and subsequently used for genotyping additional 1302 recessive individuals (Figure 5a). However, because the locus was too close to the centromere, we failed to locate it more precisely. The single recessive *lm212-1* locus was finally mapped within a region of 420 kb between the markers LD1 and RM7075, which included 57 open reading frames (ORFs). By sequencing and comparing the ORFs cloned from WT and the *lm212-1* mutant, we found a single A insertion in the third exon of *LOC_Os01g26920*, causing an early termination of translation (Figure 5b).

To assess whether *LOC_Os01g26920* was responsible for the lesion mimic phenotype, we constructed both the complementation and overexpression plasmid. The complementation one had an 8357-bp sequence, including the whole genomic DNA sequence with the 2547-bp upstream and the 1282-bp downstream sequence, while the overexpression one included the full-length coding DNA sequence (CDS) of *OsPHD1*. We obtained both transgenic lines from the transformation of the *lm212-1* mutant with these two constructs, and, as expected, these lines displayed the WT phenotype (Figure 5c,e). At the same time, we carried out the expression analysis of the *OsPHD1* gene using the qRT-PCR assay, while the expression level of OE1/2 was significantly up-regulated (Figure 5d). Overall, these results indicated that the loss of function of *OsPHD1* was the cause of the lesion phenotype of the *lm212-1* mutant.

### 2.7. Expression Patterns of OsPHD1

**Figure 6:**
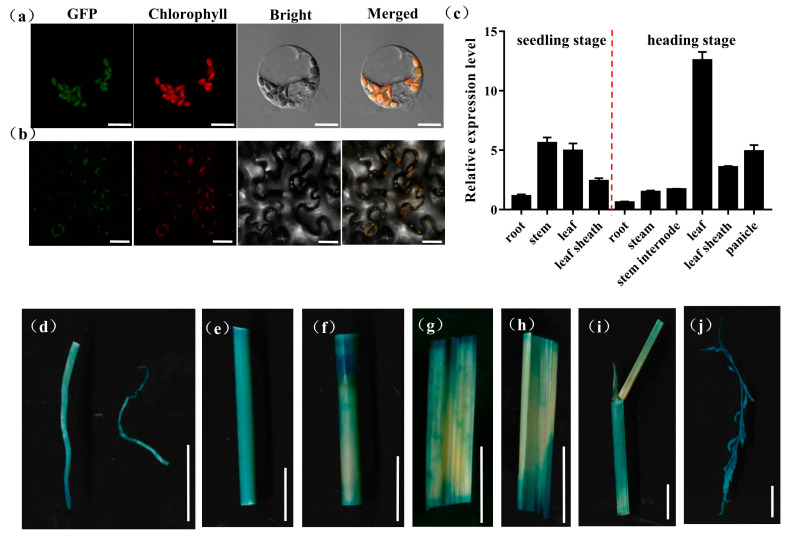
Expression patterns of *OsPHD1*. (**a**) Transcript levels of *OsPHD1* in different organs of WT at the seedling stage and the heading stage. (**b**,**c**) Subcellular localization of *OsPHD1* protein. (**b**) Rice protoplast transient assay. Scale bars, 10 μm. (**c**) *Nicotiana benthamiana* leaf assay. Scale bars, 50 μm. (**d**–**j**) Histochemical signals in plants carrying the *OsPHD1* promoter–GUS reporter gene. (**d**) root, (**e**)stem, (**f**) stem internode, (**g**,**h**) leaf, (**i**) leaf sheath, (**j**) panicle. Scale bars, 1 cm.

*OsPHD1* was annotated to function as a UDP-glucose epimerase that promotes the photosynthesis capacity in chloroplast. To further investigate the subcellular localization of *OsPHD1* protein in rice protoplasts and *Nicotiana benthamiana* leaves, the vectors expressing the OsPHD1-GFP fusion protein were generated and transiently expressed in rice protoplast and tobacco leaves, respectively. Under the confocal microscope, the green florescence signal from OsPHD1-GFP could be observed in the chloroplast, and the signal was localized with the auto-fluorescent signals of chlorophylls in the chloroplasts, which suggested that the OsPHD1-GFP fusion protein was localized in chloroplast (Figure 6a,b).

To examine the expression pattern of *OsPHD1*, we employed a qRT-PCR assay to measure *OsPHD1* expression in the root, stem, leaf, leaf sheath, panicle, and stem internode, at both the seedling stage and the heading stage. At the seedling stage, *OsPHD1* was expressed in every tested tissue and with higher expression in the stem and leaf tissue. At the heading stage, *OsPHD1* was also found in all the organs examined, and its expression was significantly higher in leaves. Moreover, the expression levels were higher in the leaf sheath and panicle than in other tissues (Figure 6c). We also generated transgenic *OsPHD1* promoter-GUS reporter. GUS staining was found in all tissues of transgenic plants (Figure 6d–j), corresponding to the qRT-PCR results. The results indicated that *OsPHD1* is constitutively expressed with the most abundance in leaves.

### 2.8. Metabolites and Phytohormone Synthesis Disorders in the lm212-1

**Figure 7:**
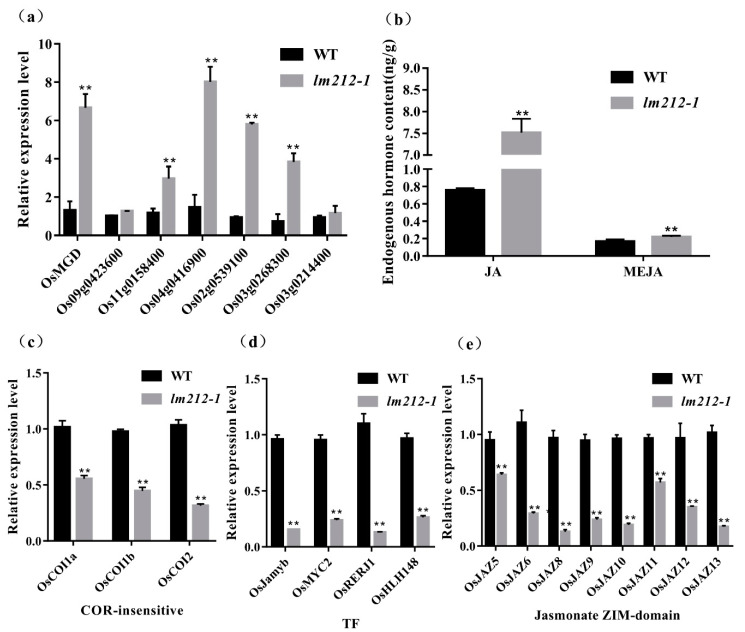
Detection of MGDG and JA-related pathway and contents of JA. (**a**) Expression analysis of MGDG synthesis-related genes in WT and *lm212-1* at the tillering stage. (**b**) Contents of JA and MeJA in WT and *lm212-1* at the tillering stage. (**c**–**e**) Expression analysis of three types of genes in the JA signal pathway. (**c**) The COR-insensitive pathway, (**d**) the TF pathway, (**e**) the jasmonate ZIM-domain pathway. ** *p* < 0.01 by the Student’s *t*-test. Data represent means ± SD of three biological replicates in (**a**,**c**,**d**). Data represent means ± SD of four biological replicates in (**b**).

*OsPHD1* encodes a UDP-glucose epimerase (UGE), which is essential for de novo biosynthesis of UDP-Gal, a precursor for MGDG biosynthesis. qRT-PCR of the MGDG biosynthesis-related genes revealed that the expression levels of *OsMGD*, *Os09g0423600*, *Os11g0158400*, *Os04g0416900*, *Os02g0539100*, *Os03g0268300*, and *Os03g0214400* were significantly up-regulated in *lm212-1* (Figure 7a). Previous studies showed that that mol% amount of MGDG was reduced in the mutant when compared to the wild type, accompanied by an increase in the abundance of other major membrane lipids, such as phosphatidylcholine (PC) [33]. PC is an important precursor for the synthesis of JA, so we determined the content of JA and MeJA. As expected, both JA and MeJA were increased in the *lm212-1* mutant (Figure 6b). We detected three types of genes in the JA signal pathway by qPCR, including COR-insensitive genes, transcription factor genes, and jasmonate ZIM-domain genes [35]. All of their expression levels were decreased in the *lm212-1* mutant (Figure 7c–e), suggesting that the self-feedback path of the JA signal pathway was activated. This effectively inhibited the JA signal pathway in a timely and effective manner to prevent plants from over-defending.

## 3. Discussion

As one of the most effective defense responses against pathogen, HR is involved in various pathways, including PCD. In LMMs, the formation of lesions originates from HR, and the defense response in most mutants is activated. Therefore, LMMs have important significance and value for the research on the mechanism of PCD and rice defense response [1]. In the present study, we identified a LMM *lm212-1* in an EMS-induced mutant bank of *japonica rice*, the mutant exhibited a lesion mimic phenotype from the seedling stage, and the lesions were temperature-sensitive and light-dependent (Figure 1).

In rice, HR involves PCD and bursts of ROS, which can inhibit the spread of pathogenic bacteria in the infected area, and is one of the most rapid and effective defense response [36]. Plants have developed a ROS detoxification system, including the nonenzymatic and enzymatic ROS-scavenging systems [5]. The nonenzymatic system oxidizes ascorbic acid to MDA and dehydroascorbate (DHA), while the enzymatic ROS-scavenging systems, including SOD, POD, ascorbate peroxidase (APX), CAT, and so on, play vital roles in removing ROS [37]. In the *lm212-1* mutant, the results of DAB staining and detection of H_2_O_2_ content both showed that H_2_O_2_ accumulated in the mutant (Figure 3a,b), and all the activities of SOD, POD, and CAT decreased (Figure 3d–f). At the same time, the contents of MDA (Figure 3g) increased, which suggested that the systems of ROS removing were disordered, and high levels of ROS appeared. Except for the excessive ROS, PCD is also accompanied by HR, and behaves in line with cell structure damage and DNA degradation [38]. As expected, Evans blue staining proved that there was cell death in the lesions in the mutant leaves (Figure 3b). All the results provided evidence that the *OsPHD1* may regulate H_2_O_2_ accumulation in the process of PCD. Many LMM mutants increased expression of defense-related genes and enhanced disease resistance to pathogens [9,39], verifying the connection between the activation of immune signals and the resistance to pathogens. The innate immune response PTI relies on pattern recognition receptors on the cell membrane surface, and interacts with effector-triggered immunity (ETI) to jointly resist the invasion of pathogens [40]. After the treatment of flg22 or chitin, the production of ROS was induced, and the dynamic accumulation rate and peak of ROS accumulation in the mutant were significantly increased compared with WT (Figure 4a,b). In order to explore whether the plant–pathogen interaction pathway was constitutively activated in the mutant or not, we detected the number of genes involved in PTI process, including *OsCEBiP* [41], *OsCERK1* [42], *OsRLCK185* [43,44], *OsBSK1* [45], *OsLysM-RLK10* [46], *OsPUB44* [47], and *OsSERK2* [48]. All the genes were significantly up-regulated in the *lm212-1* mutant (Figure 4c). To evaluate the defense response of the *lm212-1* mutant, we detected the expression levels of genes related to the defense response. The results showed that the transcripts of all these genes were upregulated in the *lm212-1* mutants compared with WT (Figure 4d). For the purpose of resistance detection, we selected three blight pathogen *Xoo* strains (CR1, PXO86, and PXO96) for inoculation (Figure 4e,f). As we expected, the resistance to *Xoo* was enhanced in the mutant. Moreover, the content of PC in the mutant was increased in the previous studies [33], and PC containing linolenic acid in *Arabidopsis* and rice became the main sources of membrane lipids for the synthesis of jasmonic acid [49]. Past research has shown that JA plays an important role in plant defense responses to pathogen infection [50]; however, the role of JA signaling in defense responses in rice is still not clear. To further explore the relationship between the mutant and the JA pathway, we detected the contents of JA and MeJA. As expected, both of them were increased (Figure 5b). Although plants can transmit through activating signal pathways and can cascade to amplify signals to achieve defense response [51], if it cannot be terminated in time, it will cause excessive trumpeting of plants, so we detected related genes in the JA signal pathway, transcription factor genes, and jasmonate ZIM-domain genes. All of their expression levels were decreased in the *lm212-1* mutant (Figure 6c–e), which showed that the self-feedback path of the JA signal pathway was activated. In summary, all of the results further verified that the loss of function of *OsPHD1* leads to the auto-activation of innate immunity, increased resistance to bacterial blight, JA content accumulation, and signal pathway disorder. Therefore, we can link rice disease resistance to the jasmonic acid pathway to a certain extent, but the role of JA in the rice defense response needs to be further elucidated in future studies.

Past research has shown that mutation of a UDP-glucose-4-epimerase results in a heightened sensitivity of root tissues to both ethylene and auxin in *Arabidopsis* [52]. In rice, OsUGE1 expression was up-regulated after drought, as well as salt or UV irradiation stress [53]. Heterologous-overexpressing BrUGE1 in rice enhanced tolerance to both salt and bacterial blight [54]. Our findings verified that the loss of function of *OsPHD1* increased resistance to bacterial blight, indicating that UDP-glucose epimerases responds to both biotic and abiotic stress in plants. As essential epimerases, UGEs are responsible for UDP-galactose synthesis, maintain sugar homeostasis in chloroplasts, which is involved in plant defense against pathogen attack [55]. Therefore, it would be worth further dissecting plant–pathogen interactions in the mutant, which may provide further insights into the underlying mechanisms of the immunity in plants.

## 4. Materials and Methods

### 4.1. Plant Materials and Growth Conditions

The *lm212-1* mutant was obtained from an EMS-induced mutant bank of a *japonica* rice, JiaHe 212. Furthermore, an F_2_ population from a cross between *lm212-1* and an indica rice Zhonghui8015 was used for genetic analysis. All plants were kwpt under the natural conditions, and were grown in a paddy filed at the China National Rice Research Institute (CNRRI) in Hangzhou, the Zhejiang province.

### 4.2. Histochemical Assay

The leaves were taken at the seedling stage for histochemical assays. Evans blue staining was used to evaluate cell death, 3,3′-diaminobenzidine (DAB) staining was used to detect H_2_O_2_ accumulation, and GUS staining was used for the spatio-temporal and tissue expression specificity assay for *OsPHD1*. These histochemical assays were performed in the same way as previous studies [24].

### 4.3. Physiological Measurements

The physiological measurements, including the SP content; H_2_O_2_ content; and activities of the POD, SOD, and MDA, followed protocols provided by the determination kit (Nanjing Jiancheng Bioengineering Institute, Nanjing, China). All the samples were measured with three biological replicates.

### 4.4. Map-Based Cloning

For gene cloning, an F_2_ population was obtained from a cross between the *lm212-1* mutant and the rice restore line Zhonghui8015. Individuals with lesion leaves from the F_2_ population were selected for gene mapping. Twenty individuals with the phenotype of *lm212-1* were initially used for linkage analysis, and 1302 F_2_ individuals with the lesion phenotype were used for fine-mapping. InDel makers surrounding the linkage region were designed according to the genomic polymorphism between the *Indica* cultivar 9311 and the *Japanica* cultivar Nipponbar for gene fine mapping. The primers sequences are listed in Table S1.

### 4.5. Vector Construction and Rice Transformation

The complementation vector *pCAMBIA1300-OsPHD1* (*PHD1-COM*) was constructed by inserting an 8357-bp genomic fragment from WT into the *Hind*III site of pCAMBIA1300 vector, using the infusion advantage cloning kit (Clontech, Beijing, China). The overexpression vector *pCAMBIA2300-OsPHD1(PHD1-OE)* was constructed by inserting the whole CDS from WT into the *Sal*I site of the pCAMBIA2300 vector using the infusion advantage cloning kit (Clontech, Beijing, China). The reporter construct *pCAMBIA1305-OsPHD1* (*PHD1-GUS*) was created by amplifying a 2596-bp fragment upstream of the ATG-starting codon of the whole CDS from WT, and was cloned into the pCAMBIA1305 binary vector (*EcoR*I and *Nco*I sites) using the infusion advantage cloning kit (Clontech, Beijing, China). The constructed recombinant vectors were introduced into the *lm212-1* mutant by *Agrobacterium tumefaciens*-mediated transformation, as previously described [56]. The primers used for these constructs are listed in Table S1. All the constructs were verified by sequencing.

### 4.6. Chlorophyll Content Investigation

The fresh leaves were collected from *lm212-1* and WT at the tillering stage for the Chl content assay. Briefly, fresh plant leaves were cut into 2-cm pieces (remove the veins) and soaked in 80% acetone, after being incubated in the dark for 24 h. The chloroplast pigment extract was measured with 80% acetone as the control, and the absorbance was measured at the wavelength of 645, 470, and 663 nm, using a microplate reader Infinite M200 PRO (TECAN, Männedorf, Switzerland). Three biological replicates were measured for each sample.

### 4.7. Photosynthesis Related Indicators Assay

In the natural environment, the photosynthetic parameters of the *lm212-1* and WT flag leaves at the heading stage were measured with a portable photosynthesis instrument Li-6800 (LI-COR, Hainesport, NJ, USA) from 9 to 10 am on a clear day, mainly including net photosynthetic rate, transpiration rate, and intercellular CO_2_ concentration. All measurements were performed under saturated irradiance. Three biological replicates were measured for each sample.

### 4.8. Evaluation of Bacterial Blight Resistance

Three *Xoo* strains (Chinese strain *CR4*, Philippine strains *PXO86* and *PXO96*) saved in this laboratory were selected to test the bacterial blight *Xoo* resistance. Each genotype was inoculated by the leaf cutting method in 10 full-spread leaves. The length of the lesions was measured 14 days after inoculation. The average lesion length of the leaves was used for comparative analysis, and the incidence of the leaves was scanned and recorded.

### 4.9. Subcellular Localization Assay

The CDS of *OsPHD1* without the stop codon was amplified and inserted into the EcoR1 site of the *pYBA1132* vector [57]. The vector was transformed into rice protoplasts and *Nicotiana benthamiana* leaves. The method of rice protoplasts preparation and plasmid transformation was carried out, as described previously [58]. Fluorescence signals were observed with a ZEISSLSM 700 (ZEISS, Jena, Germany) laser scanning confocal microscope 48 h after transfection in rice protoplasts or 72 h after infiltration in *Nicotiana benthamiana* leaves. Primers used for vector construction are listed in Table S1.

### 4.10. Measurement of ROS

ROS generation dynamics after PAMPs treatment (flg22 and chitin) were determined using the luminol chemiluminescence assay, as described previously [22]. In brief, the leaf disks (0.5 cm in diameter) were cut from WT and the *lm212-1* mutant at the tilling stage. After being soaked in sterile distilled water overnight, three leaf disks were added into a 1.5 mL microcentrifuge tube containing 8 nM of chitin or 100 nM of flg22, 1 μL of horseradish peroxidase (Jackson Immuno-Research 016-030-084), and 100 μL of luminol (Bio-Rad Immun-Star horseradish peroxidase substrate 170-5040). The sterile distilled water was used as a control. The luminescence was promptly detected every 10 s for 20 min in a Glomax 20/20 luminometer (Promega, Beijing, China). Each measurement was performed with three biological replicates for each sample.

### 4.11. RNA Extraction and qRT-PCR

The total RNA of rice leaves was extracted with the TIANGEN RNAprep pure plant kit (Tiangen Biotech, Beijing, China). Reverse transcription and qRT-PCR were performed, as described previously [24]. The rice ubiquitin gene (*LOC_Os03g13170*) was used as an internal control (primer pair Ubi), and the 2^−ΔΔCT^ method was used to calculate the relative levels of gene expression [59]. The primers used are listed in Table S1.

## 5. Conclusions

*OsPHD1* encodes a UDP-glucose epimerase, which is essential for the bioconversion of UDP-Gal and UDP-Glc. The impaired function of *OsPHD1* lead to spontaneous cell death, auto-activated defense response, and enhanced bacterial blight resistance, which may result from interrupted MGDG biosynthesis and highly accumulated JA.

## Data Availability

The data presented in this study are available on request from the corresponding author.

## Supplementary Materials

The following are available online at: https://www.mdpi.com/article/10.3390/ijms23020751/s1.
