# Supplementary material for: Disruption of OsPHD1, Encoding a UDP-Glucose Epimerase, Causes JA Accumulation and Enhanced Bacterial Blight Resistance in Rice
Source: Int J Mol Sci. 2022 Jan 11;23(2):751. doi: 10.3390/ijms23020751 (PMC8775874; doi:10.3390/ijms23020751)
Supplement: Supplementary file 1 [file ijms-23-00751-s001.zip › ijms-1524258-supplementary.pdf]

International Journal of
Molecular Sciences

MDPI

Article

Disruption of *OsPHD1*, Encoding a UDP-Glucose Epimerase, Causes JA Accumulation and Enhanced Bacterial Blight Resistance in Rice

Yu Gao [1,†], Xiaojiao Xiang [1,†], Yingxin Zhang [1], Yongrun Cao [1], Beifang Wang [1], Yue Zhang [1], Chen Wang [1], Min Jiang [1], Wenjing Duan [1], Daibo Chen [1], Xiaodeng Zhan [1], Shihua Cheng [1], Qunen Liu [1,*] and Liyong Cao [1,2,*]

[1] State Key Laboratory of Rice Biology and Chinese National Center for Rice Improvement, China National Rice Research Institute, Hangzhou 311401, China; 15057067488@163.com (Y.G.); xiangxiaojiao1990@163.com (X.X.); zhangyingxin@caas.cn (Y.Z.); caoyongrun93@163.com (Y.C.); nxywbf@163.com (B.W.); zhangyuerice@163.com (Y.Z.); 98481037wc@gmail.com (C.W.); jiang_min@webmail.hzau.edu.cn (M.J.); 13140230176@163.com (W.D.); cdb840925@163.com (D.C.); zhanxiaodeng@caas.cn (X.Z.); chengshihua@caas.cn (S.C.)

[2] Northern Center of China National Rice Research Institute, China National Rice Research Institute, Shuangyashan 155100, China

* Correspondence: liuqunen@caas.cn (Q.L.); caoliyong@caas.cn (L.C.); Tel.: +86-0571-6337-0218 (Q.L.); Tel.: +86-0571-6337-0329(L.C.)

† These authors contributed equally for this work.

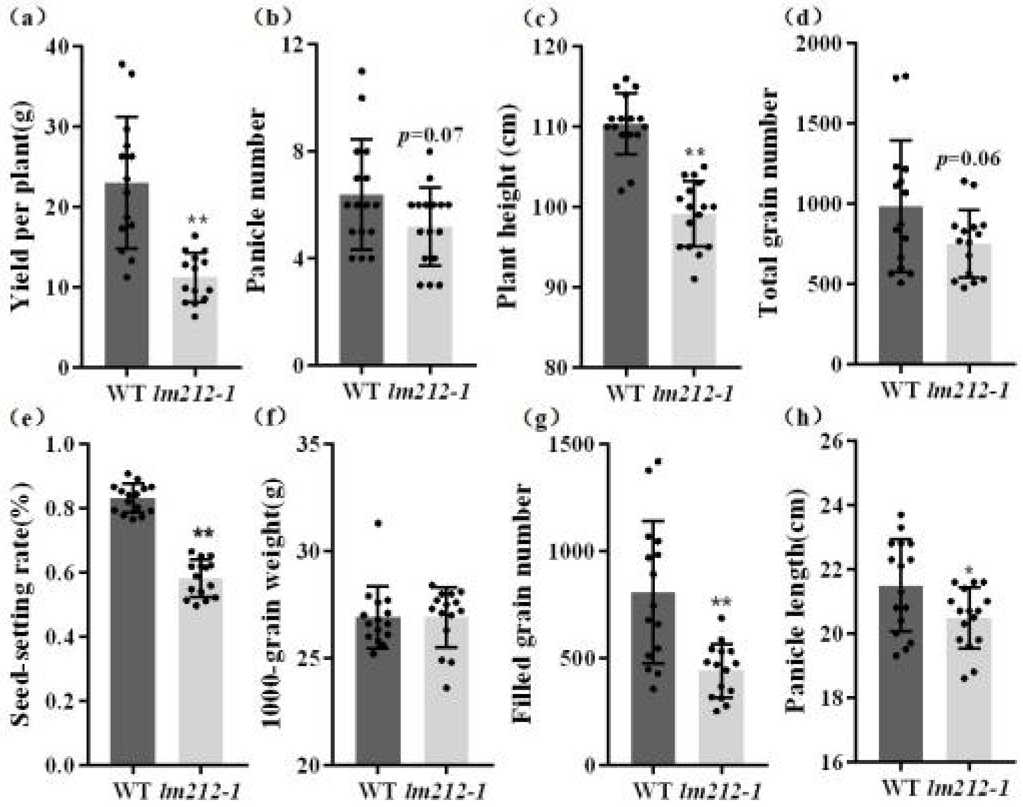

Figure S1. Several agronomic traits of WT and *lm212-1* mutant. **$p < 0.01$ by student's t-test. Data are means±SD of sixteen biological replicates.

Table S1. List of primers and their uses.

Primer name	Sequence(5′-3′)	purpose
LD1-F	TATCGACAAGTCATCTGGTG	mapping
LD1-R	GTTTCCTTGCATTACAGGTG	
LD9-F	CATTGTATATGCCCTTGTGC	
LD9-R	GCCAACAGGCAGTAATGTAT	
LD12-F	AAGGCGCTAAAAGAAACCTA	
LD12-R	GTGCAGGAAACGATTGAGAT	
LD13-F	CCTGCAGCATATATAGAGTA	
LD13-R	TGGTTTCCTTTCATATTCCG	
COM-F	TCTAGAGTCGACCTGCAGCAGGACAGTCCAGCAACGAG	complementation
COM-R	AAGCTTGCATGCCTGCAGGAGTGTACGGCGTCTTGCTT	
OE-F	ATCCTCTAGAGTCGACATGGGCGGCGCCGCCGTC	overexpression
OE-R	ATGCCTGCAGGTCGACTCAACTGTCAAGACCAAGGAATTCTG	
GUS-F	GCAGGCATGCAAGCTTAACCTACTACTCCACCTGCCA	GUS
GUS-R	CTCAGATCTACCATGGCGCGCGGCGGGCGGTGGT	
GFP-F	CGGGCTGCAGGAATTCATGGGCGGCGCCGCCGTCTC	GFP
GFP-R	GCTTGATATCGAATTCACTGTCAAGACCAAGGAATTCTGTGA	
OsUBQ10-F	TGGTCAGTAATCAGCCAGTTTGG	qPCR
OsUBQ10-R	GCACCACAAATACTTGACGAACAG	
OsPR10-F	CAGTGGTCAGTAGAGTGATCAG	
OsPR10-R	GGGTTAAGCTTCATGGTGTAGA	
OsPAL1-F	GACCCTGTATTTTCTTCGTTCG	
OsPAL1-R	AGTAGCAATACTTTCACCCCAA	
OsWRKY45-F	CGTCGACCAGATTCTCCACACCT	
OsWRKY45-R	CTCGCTCTGCACCTCCGACCC	
OsPO-C1-F	ATAGCAATGTGTACGTGGAGAT	
OsPO-C1-R	ATTCCATGCACATACAGATGGA	
OsSGR-F	AGGGGTGGTACAACAAGCTG	
OsSGR-R	GCTCCTTGCGGAAGATGTAG	
OsNYC1-F	CATGCAACACCAACAAAAGG	
OsNYC1-R	GACCATTCCAGGAGAAGCAG	
OsNYC3-F	TCTATCTAGGTGCCAAAGGC	
OsNYC3-R	ATTCTGGCACCTGCTGTTTC	
OsNOL-F	CCACGAAAGGTATAGGATATG	
OsNOL-R	TCAAGTCAGTCACCGCAGAT	
OsCOI1a-F	TACCTTCCTATCTATCCCTGGG	
OsCOI1a-R	TAGTAGCTTATTCGGGCACAAT	
OsCOI1b-F	TAAGGAGTTGTTGCTTCAGTGA	
OsCOI1b-R	CATGAGATCGTGACCAGTCTTA	
OsCOI2-F	TGCTACCAACAATAGTGTCCTT	

OsCOI2-R	CTTCAAAACTTTCAGCCTTCGA
OsJamyb-F	GTTGATGATAGCGGAGACTACA
OsJamyb-R	TCTTCAAATCTTGTGACTCCGA
OsMYC2-F	GGGTTTTGTTATCTCCTCTCCT
OsMYC2-R	AGAATAGGAGGAGAGGAAAGGT
OsRERJ1-F	GGATAGTGTCCAAATGAAGCAG
OsRERJ1-R	CCAAAACGCATGACTCCATATA
OsbHLH148-F	CACAGACATCTCATGTGCAAAA
OsbHLH148-R	CTTGCTCCATCACAAGCTTTTA
OsJAZ13-F	ACACGTCAGCTTTAATCCCATA
OsJAZ13-R	AGGAATAATCGTGCACTGTACA
OsJAZ12-F	ACCCAAAGAGTTCTTTCACTGA
OsJAZ12-R	TGGAGACAGTTGTACTTGTACA
OsJAZ11-F	TGAGCCGAAGAAACTGAAGATA
OsJAZ11-R	ACAGACACGTGGAAATCAAATG
OsJAZ10-F	CGCAAGAGGATTGATTCACTTT
OsJAZ10-R	TTCCCATCAATTCAATCTCCCT
OsJAZ9-F	AGAATTGAGAGCTCGATCGATT
OsJAZ9-R	AATCAACTCAAGGCCGTTAATG
OsJAZ8-F	ATGAATGCAAATGCTCCATACC
OsJAZ8-R	GCTTTATTGTCATCTCTTGGCC
OsJAZ6-F	GAGCCAATGAATCAATGCTCAA
OsJAZ6-R	CATCGGATCATCTCCTAACACA
OsJAZ5-F	ATTACTGTTGATTTGGTCCCCT
OsJAZ5-R	CGATCGATCAACACTAAACAGG
OsCEBiP-F	TTCATCAATCAGCGATACCTCA
OsCEBiP-R	CTGGTTTGTATGATGAGCGATG
OsCERK1-F	TGAACAACCTGTATGAGAACCA
OsCERK1-R	ATGTACAATTTTCCCAGTGTGC
OsRLCK185-F	ACTGGGTCGAATTAGGTACATG
OsRLCK185-R	ATTTGCATGATCAATCACGAGG
OsBSK1-F	ACCTGGCATATACACCTCCA
OsBSK1-R	TGTCAAGTGCAAGGGTAGGA
OsLysM-RLK10-F	GTTAGCGAATCAAAAGGACTGG
OsLysM-RLK10-R	GATCAATCAGTTCGTCAAGAGC
OsPUB44-F	ATGCCTTACTGAAACCAAAACC
OsPUB44-R	ATTCATAGCAAACCCCGAGTAG
OsSERK2-F	GGCTATGGAATCATGCTTCTTG
OsSERK2-R	CAATCGAGCAACATCACATCAT
OsMGD-F	TTTTCATACCATCGCCAGAAAC
OsMGD-R	TAGGACAAAGGCAAATTTCACG
Os09g0423600-F	ACCAGGTATTCGTCACTGATTT
Os09g0423600-R	AGTATGTCATCTTCCACAAGGG
Os11g0158400-F	AGAACGGAGAAGCATAATTCCT
Os11g0158400-R	GGCTCTTCCAAAATAGCAATGT
Os04g0416900-F	GGTTATTCTGGACAAGATGGGA
Os04g0416900-R	GATCTCATTGCCAGTTAAGCAG
Os02g0539100-F	CTGAACTGGTATCATCATGGGA
Os02g0539100-R	ATTGTTGATGTGTTTGACCAGG

Os03g0268300-F	GCATTCTTCAAGGGTGCATATT
Os03g0268300-R	CAGCTCCATCTTAAGACCAGAT
Os03g0214400-F	ATAAGTTCAGGCGAGTGATAGG
Os03g0214400-R	AATCTTGTGGCAATAAATCCGG
Os01g26920-F	GTTCTCGGGGTGTCTGATGATA
Os01g26920-R	CCAACAATCAGCAAATCGTTCT

Table S2. Genetic analysis of the *lm212-1* mutant.

Cross combination	F_1 phenotype	Segregation in F_2		$\chi 2$
		Wild type	Mutant type	
*lm212-1*xZhongHui8015	Wild type	586	214	0.64
